# 3D Ultrastructure of Synaptic Inputs to Distinct GABAergic Neurons in the Mouse Primary Visual Cortex

**DOI:** 10.1093/cercor/bhaa378

**Published:** 2020-12-22

**Authors:** Yang-Sun Hwang, Catherine Maclachlan, Jérôme Blanc, Anaëlle Dubois, Carl C H Petersen, Graham Knott, Seung-Hee Lee

**Affiliations:** 1 Department of Biological Sciences, KAIST, Daejeon, 34141, Republic of Korea; 2 Biological Electron Microscopy Facility, Faculty of Life Sciences, École Polytechnique Fédérale de Lausanne (EPFL), Lausanne, CH-1015, Switzerland; 3 Laboratory of Sensory Processing, Brain Mind Institute, Faculty of Life Sciences, École Polytechnique Fédérale de Lausanne (EPFL), Lausanne, CH-1015, Switzerland

**Keywords:** excitation–inhibition balance, GABAergic interneurons, perisomatic synaptic inputs, primary visual cortex, serial block-face scanning electron microscopy

## Abstract

Synapses are the fundamental elements of the brain’s complicated neural networks. Although the ultrastructure of synapses has been extensively studied, the difference in how synaptic inputs are organized onto distinct neuronal types is not yet fully understood. Here, we examined the cell-type-specific ultrastructure of proximal processes from the soma of parvalbumin-positive (PV^+^) and somatostatin-positive (SST^+^) GABAergic neurons in comparison with a pyramidal neuron in the mouse primary visual cortex (V1), using serial block-face scanning electron microscopy. Interestingly, each type of neuron organizes excitatory and inhibitory synapses in a unique way. First, we found that a subset of SST^+^ neurons are spiny, having spines on both soma and dendrites. Each of those spines has a highly complicated structure that has up to eight synaptic inputs. Next, the PV^+^ and SST^+^ neurons receive more robust excitatory inputs to their perisoma than does the pyramidal neuron. Notably, excitatory synapses on GABAergic neurons were often multiple-synapse boutons, making another synapse on distal dendrites. On the other hand, inhibitory synapses near the soma were often single-targeting multiple boutons. Collectively, our data demonstrate that synaptic inputs near the soma are differentially organized across cell types and form a network that balances inhibition and excitation in the V1.

## Introduction

Synaptic integration is the basic function of the neural network. Synaptic inputs can be broadly classified as excitatory and inhibitory and are fundamental elements of the brain’s complicated neural networks. In the mammalian cortex, the balance between excitatory and inhibitory inputs (E/I balance) is critical for information processing. For example, in the sensory cortex, the E/I balance must be maintained to shape adequate neural responses to external sensory stimuli (Isaacson and Scanziani 2011; Tao et al. 2014; Xue et al. 2014; Kim et al. 2017). Impairment of the E/I balance results in the dysfunction of sensory processing, which has been observed in neurodevelopmental disorders such as autism and schizophrenia (Yizhar et al. 2011; Selimbeyoglu et al. 2017; Ferguson and Gao 2018).

A neuron integrates thousands of synaptic inputs that either excite or inhibit the membrane potential of the neuron. During the integration process, the location of the synaptic inputs in relation to the soma is critical, because their impact on the somatic post-synaptic potential decreases as the distance from the soma increases (Stuart and Spruston 1998; Krueppel et al. 2011). The morphological properties of the post-synaptic membrane also determine how these inputs affect membrane potential (Terzuolo and Araki 1961; Spruston et al. 1994; Nimchinsky et al. 2002). For instance, spines are specialized protrusions of the membrane that can amplify the post-synaptic potential by enriching the post-synaptic density with receptors (Miller et al. 1985; Nimchinsky et al. 2002; Harnett et al. 2012). Dendritic spines in the pyramidal neurons receive mostly excitatory synaptic inputs (Glantz and Lewis 2000). Inhibitory synaptic inputs reside not only on the dendritic shaft but also on the spine (Chen et al. 2012; Kubota et al. 2016; Villa et al. 2016). However, whether excitatory and inhibitory synaptic inputs are distributed equally across neuronal types is unknown.

The absence of spines has traditionally been considered one of the most prominent features of the inhibitory interneurons (Kwan et al. 2012). However, in recent studies, spines were observed on inhibitory interneurons in the hippocampus (Guirado et al. 2014; Scheuss and Bonhoeffer 2014) and the cortex (Buhl et al. 1997; Kawaguchi et al. 2006; Keck et al. 2011; Sancho and Bloodgood 2018). The spines on the cortical interneurons were enriched with the α-amino-3-hydroxy-5-methyl-4 isoxazole propionic acid (AMPA) and the N-methyl-D-aspartate (NMDA) receptors (Scheuss and Bonhoeffer 2014; Sancho and Bloodgood 2018). Such enrichment suggests that the function of spines in interneurons is similar to that of spines in pyramidal neurons, namely, to receive excitatory inputs. Furthermore, the number of spines on interneurons in the visual cortex decreased when adult mice were deprived of visual experience by the introduction of focal lesions in the retina (Keck et al. 2011). These data indicate that the spines of inhibitory interneurons are like those of pyramidal neurons in the cortical network and can be remodeled through activity. Furthermore, a recent study has shown that the density of spines on the dendrites of PV^+^ neurons is low but consistent (Sancho and Bloodgood 2018). Still, a definitive functional and morphological comparison of spines among different subtypes of GABAergic interneurons has yet to be conducted.

Research on gene-expression patterns and synaptic connectivity has identified distinct classes of GABAergic interneurons in the cortex (Connor and Peters 1984; Meinecke and Peters 1986; Gonchar and Burkhalter 1997; Kawaguchi and Kubota 1997; Isaacson and Scanziani 2011; Pfeffer et al. 2013). In particular, the calcium-binding protein parvalbumin (PV) and the neuropeptide somatostatin (SST), the key molecules that define distinct GABAergic neurons, account for nearly 70% of the entire population of the GABAergic neurons in the cortex (Rudy et al. 2011). Interestingly, PV^+^ neurons are known to be fast-spiking, whereas SST^+^ neurons show either burst spiking or regular spiking (Kawaguchi et al. 1987; Kawaguchi and Kubota 1996; Wamsley and Fishell 2017). Short-term synaptic plasticity of excitatory synaptic input is another robust feature differentiating PV^+^ and SST^+^, with only SST^+^ neurons receiving prominent facilitating glutamatergic input *in vitro* (Reyes et al. 1998) and *in vivo* (Pala and Petersen 2015). Furthermore, PV^+^ neurons strongly inhibit peri-somatic regions of excitatory pyramidal neurons, whereas SST^+^ neurons inhibit the distal dendrites of these neurons (Taniguchi et al. 2011). Even within the same class of GABAergic neurons, different physiological and synaptic properties have been observed. For example, there are two types of SST^+^ neurons in the rat hippocampus, bistratified and O-LM neurons, both of which have distinct axonal arbors and theta oscillation frequencies (Katona et al. 2014). In the mouse somatosensory cortex, SST^+^ neurons show various physiological features that can be used to further classify the sub-classes of SST^+^ neurons (Munoz et al. 2017; Nigro et al. 2018). However, it is still unclear whether the synaptic inputs of these subtypes of interneurons are organized in distinct ways.

In this study, we examined PV^+^ and SST^+^ neurons in layer 2/3 of V1 in transgenic mouse lines that express fluorescent proteins in a subset of GABAergic neurons using serial block-face scanning electron microscopy (SBEM) through correlative light and electron microscopy (CLEM) (Maclachlan et al. 2018). In particular, we examined the peri-somatic structure in different types of GABAergic neurons to understand how the synaptic inputs are integrated near the soma. We first examined the ultrastructure of somatic membranes and their protrusions, including dendrites, axons, primary cilia and spines. We found that whereas SST^+^ neurons were divided into two classes, “spiny” and “aspiny,” PV^+^ neurons were almost always “aspiny” with a low density of spines. Moreover, we examined putative excitatory (type I, asymmetrical) and inhibitory (type II, symmetrical) synapses in each cell type and found that the ratios of excitatory and inhibitory inputs differed between cell types. Furthermore, we found different patterns of synaptic inputs across cell types: excitatory synapses on GABAergic neurons were often multiple-synapse boutons (MSBs), which have multiple post-synaptic targets from a single presynaptic bouton, and perisomatic inhibitory synapses were the single-targeting multiple boutons (STMBs), which have multiple boutons from the same axon on the same target neuron. Our data illustrate the ultrastructure of soma and proximal dendrites of specific target neurons and demonstrate how the synaptic structures are organized in different types of inhibitory interneurons.

## Materials and Methods

### Animals

The Swiss Federal Veterinary Office and the Canton of Vaud Veterinary Office (license number VD1628) approved all experimental procedures for animal usage. All experiments were performed according to the guidelines of the Swiss Federal Act on Animal Protection and Swiss Animal Protection Ordinance. We used PV-Cre mice (The Jackson Laboratory, #008069, B6;129P2-Pvalb^tm1(cre)Arbr^/J) (Hippenmeyer et al. 2005) and SST-Cre mice (The Jackson Laboratory, #013044, STOCK Sst^tm2.1(cre)Zjh^/J) (Taniguchi et al. 2011) crossed with ROSA-LSL-tdTomato mice (Ai14, The Jackson Laboratory, #007914, B6.Cg-Gt(ROSA)26Sor^tm14(CAG-tdTomato)Hze^/J) (Madisen et al. 2010) to label PV^+^ and SST^+^ neurons with tdTomato (PV::tdTomato and SST::tdTomato). To label a subset of SST^+^ neurons, we used GIN mice (The Jackson Laboratory, #003718, FVB-Tg(GadGFP)45704Swn/J) (Oliva Jr. et al. 2000).

### Tissue Preparation

We followed previous protocols for tissue preparation (Maclachlan et al. 2018). Adult mice (male and female; 8–12 weeks of age) were anesthetized with an overdose of inhaled anesthetic (isoflurane, Terrel) before euthanization. Mice then underwent cardiac perfusion of a solution of 2% paraformaldehyde (Electron Microscopy Sciences, 15714) and 2.5% glutaraldehyde (Electron Microscopy Sciences, 16220) at pH 7.4. The brains were then extracted and embedded in 5% agarose gel cube, and 80 micron-thick, coronal sections of the brain around V1 regions were cut with a vibratome (Leica, VT1200S).

### SBEM Imaging

We performed SBEM imaging, as described earlier (Maclachlan et al. 2018). We first identified the target area in layers 2 and 3 (L2/3) of V1 under bright-field illumination of the coronal section of the mouse brain to identify natural landmarks in the slice, such as the boundary between brain slices and blood vessels, using an upright light microscope (Leica). Next, we obtained images near the soma of red-fluorescent PV^+^ neurons, red-fluorescent SST^+^ neurons, or green-fluorescent GIN neurons in an area within L2/3 of V1 sections of PV::tdTomato, SST::tdTomato or GIN mice, using a confocal microscope (SP8 STED 3X, Leica). We then imaged the same area of target cells with a custom-built two-photon microscope (Avermann et al. 2012), and with its laser, we burned two vertical lines and one horizontal line around the desired neuron. This marked region was 80–100 μm wide and 20–30 μm deep. We reconfirmed the location of the target neuron and laser marks using both a confocal microscope to obtain z-sectioned images (300-nm intervals, total 50–60 μm along the z-axis) and a bright-field microscope (Leica). The imaged brain slice was then stained and embedded for electron microscopy following the previously reported protocol (Hua et al. 2015). Once the resin had cured, the region of the slice containing the region of interest was glued to an aluminium sample holder (Gatan, Inc., Pleasanton, CA, USA) and trimmed with a glass knife mounted in an ultramicrotome (Leica Microsystems, UC6) until the block face measured approximately 250 × 250 μm. Next, the sample was coated with a 50-nm layer of gold sputter (Quorum Technologies; Q300T), placed inside a scanning electron microscope (Merlin, Zeiss NTS) fitted with an ultramicrotome (3View system, Gatan, Inc., Pleasanton, CA, USA), and left overnight for the vibrations to stabilize before the start of the serial cutting and imaging process. To obtain serial SBEM images with appropriate resolution and window size, we collected images with a pixel size of 5.1 nm, with an image size of 6000 × 6000 pixels (∼70 MB/image), and 50-nm section thickness up to 600 sections.

### Image Processing, 3D Reconstruction, and Analysis

All alignment and quantitative analyses of the serial SBEM images were carried out using the TrakEM tools (Cardona et al. 2012) in the FIJI software (http://fiji.sc/). To reconstruct the perisomatic membrane and input structures in the aligned images, we first identified the soma of the target cells. We then tracked protrusions from the soma manually in the FIJI software. Each compartment was colored and sorted into a different list: soma, axons, dendrites, spines, or excitatory or inhibitory pre-synaptic structures. The structures of the excitatory or inhibitory synapses were identified by the symmetry between pre-synaptic and post-synaptic membranes and by the shape of synaptic vesicles in the pre-synaptic bouton (Korogod et al. 2015). If the pre- and post-synaptic structures were asymmetrical, and the vesicles were large and circular, the synapses were classified as excitatory synapses (type I synapses). If the pre- and post-synaptic structures were symmetrical, and the vesicles were small and oval, the synapses were counted as inhibitory synapses (type II synapses). To measure the size of synapses, we randomly selected up to 10 from each type of synapse that was located in a distinct compartment across neuronal types. We then measured the 2-dimensional sizes of the synaptic junction areas as shown previously (Kubota et al. 2015). All traced structures were reconstructed with resample values of 1 to 3 to maintain the best resolution poissible and then exported as a wavefront format (.obj) for the next analysis, using Blender software (https://www.blender.org/).

In Blender, all imported structures underwent a “remesh” modification to optimize the mesh structure and smooth the outer surface of the object (mode: smooth, octree depth: 8–10, scale: 0.90–0.99). To measure the volume and surface area of the soma, we created an icosphere consisting of 80 triangles and adjusted the icosphere to cover the reconstructed soma completely. The volume and surface area of the icosphere were measured by the 3D printing add-ons (freely accessible in Blender). To measure the length and diameter of neurites and spines, we used the NeuroMorph software toolset to the mesh models in Blender, and the centerline of each protrusion was computed by the VMTK (Vascular Modeling Toolkit; http://www.vmtk.org/) as shown previously (Antiga and Steinman 2004; Antiga et al. 2008; Jorstad et al. 2018). The centerline was used to reconstruct the cross-sections across each protrusion in NeuroMorph. Cross sections were randomly picked, at least 10 points per 1-μm protrusion. The NeuroMorph software calculated maximum and minimum radiuses from the center to the surface of the cross sections that had been reconstructed as irregular circles filling the protrusion. We then calculated the diameter of each section from the median radius. The diameter of each protrusion was averaged across sections (at least 10 cross sections per 1-μm length of the protrusion).

### Statistical Analysis

All data are presented as means ± standard errors of the mean (SEM). “*N*” in the figure represents individual neurons, and “*n*” represents individual dendrites or spines. We tested normality of distribution for each dataset using the Lilliefors test. We used the Kruskal–Wallis test or One-way ANOVA test with Bonferroni correction to determine whether differences between groups were statistically significant. We used the Mann–Whitney *U* test or unpaired *T* test for unpaired comparision between two datasets. We used the Wilcoxon signed-rank test or paired *T* test for paired comparision between two datasets. Differences were considered statistically significant if *P* < 0.05. Results of the statistical tests are reported in the main text or figure captions. Throughout the paper, * indicates ^*^P < 0.05, ^**^P < 0.01, and ^***^P < 0.001. All analyses were performed in SPSS (IBM) and Excel (Microsoft).

**Figure 1:**
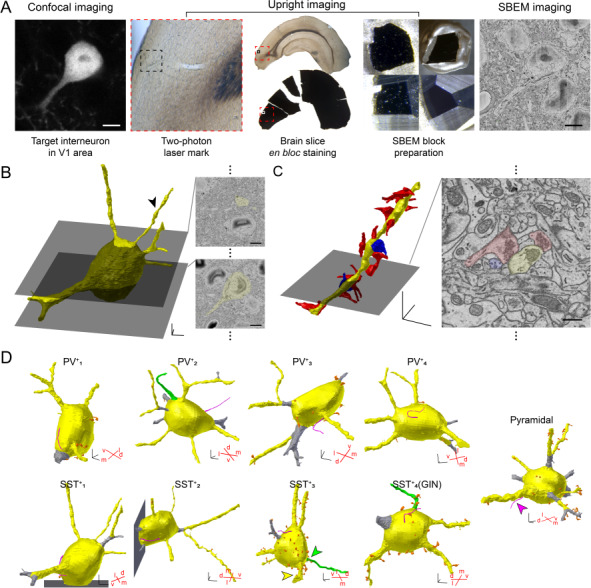
3D reconstruction of a targeted neuron in the V1. (*A*) SBEM procedure to image a targeted neuron in the V1: A brain slice containing V1 was obtained from an SST::tdTomato mouse and imaged with a confocal microscope to locate a red fluorescent SST^+^ neuron. Laser marks were made using a two-photon microscope. The slice was processed for electron microscopy and then trimmed on the pin stub for SBEM imaging. Scale bars, 5 μm; red squares, V1 in the slice; black squares, the local area in the V1 where the target neuron was located. (*B*) Reconstructed tdTomato^+^ neuron in (*A*) (left, yellow) and representative SBEM images of the neurons (right). 3D-scale bar, 3 μm; scale bars, 5 μm. (*C*) An example dendrite of the neuron in (*B*, arrowhead). Left, reconstructed excitatory (red) and inhibitory (blue) pre-synaptic inputs; right, a representative image from SBEM. 3D-scale bar, 3 μm; scale bar, 500 nm. (*D*) 3D reconstruction of the initial processes from the soma (yellow): dendrites (yellow), axons (green), cilia (magenta), and spines (orange). Dendritic segments that were shorter than 5 μm were excluded from the quantitative analysis (gray). Red crosses, ventrodorsal and mediolateral angles of the neuron in the 3D space of the brain samples. 3D-scale bars, 3 μm. Note that all identified axons (1 in PV^+^ and 2 in SST^+^) project in the dorsal direction.

## Results

### Identification of the Peri-Somatic Ultrastructure of PV^+^, SST^+^, and Pyramidal Neurons

To examine and compare the ultrastructures of specific cell types in layer 2/3 of mouse V1, we identified the fluorescence-labeled target neurons by comparing the confocal image to the serial SBEM images. The structure of the target neuron was traced in a series of images, and traced images were reconstructed as 3-dimensional structures (Fig. 1*A*,*B*; see also Movie S1). Within the 31 × 31 × 30-μm^3^ SBEM image window in each sample, there were no fluorescent cell bodies other than the target neuron. High-resolution (5.1 × 5.1 nm^2^ per pixel) images allowed the observation and reconstruction of the post-synaptic structures and pre-synaptic boutons that formed on the soma and proximal dendrites within the target cell (Fig. 1*C*).

We identified 4 PV^+^ neurons in brain samples from PV::tdTomato mice, 3 SST^+^ neurons in brain samples from SST::tdTomato mice, and 1 SST^+^ neuron in the brain sample from a GIN mouse. We also examined one putative pyramidal neuron that was not labeled with fluorescence by reconstructing one cell in the SST::tdTomato brain slice. A total of 9 traced neurons included most of the somatic and proximal membrane protrusions from the soma (Fig. 1*D*). Seven among nine samples covered the whole soma, showing that the volume of the soma of traced neurons ranged from 927.39 to 1805.11 μm^3^ (see details in Table 1). The length of proximal dendrites ranged from 2.43 to 30.13 μm, including the dendrites from two SST^+^ neurons whose somas had been partially traced (see Table 2). Using the techniques of SBEM and CLEM, we successfully identified the 3D ultrastructure of particular cell types in the intact V1 circuits. In particular, in SBEM images, we were able to examine the detailed structures of synaptic inputs.

**Table 1:** Information for neurons imaged by SBEM

Cell type	Mouse		Soma			Axon	Cilia	Number of dendrites	
	Sex	Age	Volume (μm^3^)	Surface area (μm^2^)	Number of spines			Initial only	Branches < 5 μm
PV^+^_1_	M	P69	1322.00	638.90	6	N	Y	4	4+
PV^+^_2_	F	P69	1607.36	702.28	0	Y	Y	8	10+
PV^+^_3_	F	P69	927.39	516.57	8	N	Y	4	6+
PV^+^_4_	F	P49	1529.67	684.84	6	N	Y	6	6
SST^+^_1_*	F	P89	1211.36	551.92	2	N	Y	5	6
SST^+^_2_*	M	P81	946.01	442.33	0	N	Y	5	6
SST^+^_3_	F	P89	1805.11	763.45	33	Y	Y	6	7
SST^+^_4_ (GIN)	M	P73	1431.15	660.49	13	Y	Y	4	4+
Pyramidal	M	P81	1103.31	546.78	7	N	Y	6	8+

Note: Each cell was collected from different mouse brains, as noted. Volumes and surface areas were analyzed by Blender software. ^*^Neurons with partial image stacks of soma. Y: yes (observed), N: no (not observed within the images). +: more branches may exist (in case of some branches that were shorter than 5 μm from the soma).

**Table 2:** Information for dendrites imaged in each neuron

Cell type	Initial dendritic segments	Number of branches (1st–2nd–3rd)	Number of spines	Length of initial segments (μm)	Length of total segments (μm)	Diameter of initial segments (within 5 μm) (mean ± SEM)
PV^+^_1_	1	-	3	10.62	-	1.27 ± 0.02
	2	3	-	6.21	26.18	1.47 ± 0.02
	3	-	-	11.08	-	1.16 ± 0.02
	4	-	1	2.43	-	-
PV^+^_2_	1	2–2	-	5.74	33.80	1.34 ± 0.04
	2	-	1	10.81	-	1.24 ± 0.01
	3	2	-	4.52	7.40	-
	4	-	-	13.67	-	0.81 ± 0.02
	5	-	-	3.74	-	-
	6	-	1	3.45	-	-
	7	2	1	3.84	25.25	-
	8	-	-	11.48	-	0.88 ± 0.01
PV^+^_3_	1	2	-	2.26	29.08	-
	2	2–2-2	4	3.32	29.55	-
	3	-	2	4.69	-	-
	4	-	1	17.8	-	1.03 ± 0.01
PV^+^_4_	1	-	-	9.95	-	1.35 ± 0.02
	2	2	-	8.42	13.83	1.12 ± 0.01
	3	-	-	8.97	-	1.73 ± 0.01
	4	-	1	11.71	-	1.53 ± 0.03
	5	2	1	7.40	21.90	1.38 ± 0.02
	6	2	1	5.69	9.78	1.38 ± 0.02
SST^+^_1_	1	2	-	9.50	15.92	1.91 ± 0.02
	2	-	-	16.01	-	0.57 ± 0.02
	3	2	-	3.53	15.55	-
	4	-	-	18.57	-	0.98 ± 0.02
	5	-	-	6.23	-	0.70 ± 0.01
SST^+^_2_	1	2	2	1.41	51.50	-
	2	-	-	8.03	-	0.89 ± 0.01
	3	-	-	10.18	-	0.34 ± 0.01
	4	-	1	6.75	-	0.90 ± 0.01
	5	-	-	2.54	-	-
SST^+^_3_	1	-	5	11.49	-	1.39 ± 0.02
	2	-	3	5.98	-	0.62 ± 0.01
	3	-	8	11.80	-	2.25 ± 0.03
	4	-	7	17.09	-	1.58 ± 0.02
	5	-	6	17.68	-	1.94 ± 0.02
	6	2	4	3.64	8.56	-
SST^+^_4_ (GIN)	1	2	6	8.66	20.67	2.43 ± 0.05
	2	-	5	14.79	-	1.73 ± 0.03
	3	-	-	3.50	-	-
	4	-	4	13.36	-	1.77 ± 0.04
Pyramidal	1	2–2	15	4.30	29.29	-
	2	-	3	18.56	-	2.75 ± 0.08
	3	-	-	3.16	-	-
	4	-	1	7.65	-	0.89 ± 0.01
	5	-	-	3.06	-	-
	6	2	15	4.66	30.31	-

Note: Diameters and lengths of dendrites and axons were analyzed by Blender software. Number of branches = 1st branch: branch from initial dendritic segment; 2nd branch: branch from 1st branch; 3rd branch: branch from 2nd branch.

### Membrane Protrusions from Cell Bodies

**Figure 2:**
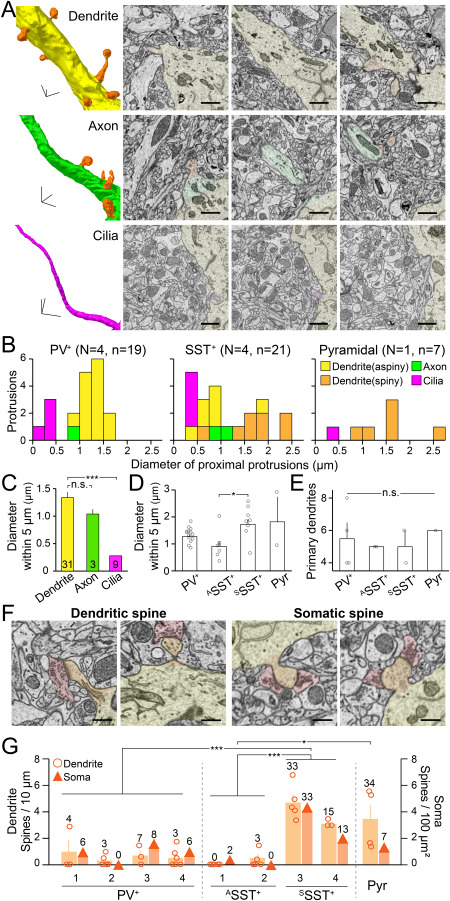
Three main processes and spines from the soma of PV^+^, SST^+^, and pyramidal neurons. (*A*) Representative 3D structures and SBEM images of the protrusions from neurons in the Fig. 1*D* (arrowheads). Different colors indicate different types of protrusions: dendrite (yellow), axon (green), cilia (magenta), and spine (orange). scale bars, 1 μm. (*B*) Histograms indicate the thickness of different types of protrusions in PV^+^ (left), SST^+^ (middle) and pyramidal neurons (right). *N*, the number of imaged neurons; n, the number of protrusions. Yellow, aspiny dendrites; orange, spiny dendrites; green, axons; magenta, cilia. Note that PV^+^ and SST^+^ neurons appear to have different distributions of dendritic thickness. (*C*) The diameter of the initial segments (5 μm) of different types of processes from the soma; dendrites (*n* = 31), axons (*n* = 3), and cilia (*n* = 9). Bars, mean ± SEM. ^***^P < 0.001; n.s., not significant; ANOVA test with Bonferroni correction. (*D*) The diameter of the initial segments (5 μm) of dendrites from different neuronal types. Bars, mean ± SEM; open circles, individual cells; n.s., not significant; ^*^P < 0.05; Kruskal–Wallis test with Bonferroni correction. (*E*) The number of initial segments of dendrites from different neuronal types. Bars, means ± SEM; open circles, individual cells; n.s., not significant; Kruskal–Wallis test with Bonferroni correction. (*F*) Representative SBEM images of spines (orange) forming synapses with excitatory pre-synaptic inputs (red) on dendrites (left) and soma (right). Scale bars, 500 nm. (*G*) Spine density on the dendrites and the soma of individual neurons. Circles, number of spines per 10 μm of each dendrite; wide orange bars, means ± SEM; triangles, number of spines per 100-μm^2^ surface of soma. ^*^P < 0.05; ^***^P < 0.001; Kruskal–Wallis test with Bonferroni correction.

We first examined the membrane protrusions extending from the soma and classified them into four categories according to the structure: dendrites, axons, cilia, and spines. We first examined the long protrusions (dendrites, axons, and cilia; Movie S2). Dendrites were found in all cells, and the number of first dendritic protrusions ranged from four to eight per cell (Figs 1*D* and 2*A*, yellow). In contrast, axons were rarely identified because the structures of their initial protrusions did not differ much from those of dendritic protrusions. It is also possible that the axons might be outside of the traced areas, as shown before that axons of interneurons often emerge from dendritic segments rather than directly from the soma (Meyer and Wahle 1988; Hofflin et al. 2017). Nevertherless, we identified three protrusions that had a few synaptic inputs at the most proximal area and no other synaptic inputs along the protrusions. These we classified as potential axons, distinguishing them from classical dendrites that had post-synaptic structures across the imaged sections (Figs 1*D* and 2*A*, green). Although we were not able to identify axons, we identified cilia in all cells imaged (Figs 1*D* and 2*A*, magenta). The structure of cilia was unique, namely, a thin and tube-like protrusion with an average diameter of about 0.27 ± 0.01 μm and an average length about 10.13 ± 0.37 μm; synaptic structures were absent (Fig. 2*A*,*B*). We were able to observe the typical microtubule structure in the cilia (Fig. 2*A*).

The diameter of dendrites was similar to that of axons but significantly thicker than the diameter of cilia (Fig. 2*B*,*C*). However, unlike the uniform thickness of the cilia and axons across cell types, the thickness of dendrites varied (Fig. 2*B*). Interestingly, the histogram of dendritic thickness showed different patterns betweeen cell types (Fig. 2*B*). PV^+^ neurons appeared to show near normal distribution in the dendritic thickness with a clear peak in the middle (Fig. 2*B*). On the other hand, the histogram of the dendritic thickness of SST^+^ neurons appeared to show two peaks: one group of dendrites showed similar or slightly thinner thickness compared to the other group (Fig. 2*B*, middle). Interestingly, in SST^+^ neurons, the thinner dendrites were aspiny and the thicker ones were spiny (Fig. 2*B*,*D*; see below). Among the dendrites of the pyramidal neuron, the thickest dendrite projected toward the dorsal side of the cortex, suggesting that it was an apical dendrite. A previous report showed that the frontal cortex of young rats has fast-spiking neurons with more primary dendrites than the SST+ neurons (Kawaguchi et al. 2006). However, in our limited data set, the various cell types in the V1 of adult mice appeared to show a similar number of primary dendrites (Fig. 2*E*), although we were not able to trace total dendritic segments across cell types due to the limitation of images that we obtained along the Z axis of the cell (Table 1).

### Number and Structure of Spines in Different Types of Neurons

Last, the spines were defined as short membrane protrusions, broadly distributed on a neuron’s soma, dendrites and axons (Fig. 2*F*). Spines had at least one post-synaptic structure, and these protrusions can be distinguished from small immature protrusions without synapses, which were rarely observed in our samples. We focused on the spine’s protrusions to understand how the synaptic inputs in each type of neuron are organized. We first measured how broadly spines were distributed in distinct cell types such as PV^+^, SST^+^, and pyramidal neurons. Some neurons had many spines, others did not. The spines on the soma and dendrites were counted and classified as dendritic and somatic. The greater the spine density on the dendrites, the more spines were found on the soma.

Interestingly, the number of spines differed across neuronal types (Fig. 2*G*). All PV^+^ neurons had sparsely distributed spines on both soma and dendrites (0.54 ± 0.19 spines per 10 μm of dendrite, and 5.00 ± 1.73 spines per soma) as shown earlier (Sancho and Bloodgood 2018). On the other hand, there appeared to be two groups of SST^+^ neurons: one showed a very low density of spines on the perisoma (0.21 ± 0.17 spines per 10 μm of dendrite and 1.00 ± 1.00 spines per soma), and the other had a similar or even higher number of spines (4.12 ± 0.46 spines per 10 μm of dendrite and 23.00 ± 10.00 spines per soma) compared with the pyramidal neuron (3.41 ± 1.13 spines per 10 μm of dendrite and 7 somatic spines). Overall, we were able to identify four distinct cell types based on the presence of spines among imaged neurons: PV^+^, aspiny SST^+^ (^A^SST^+^), spiny SST^+^ (^S^SST^+^), and spiny pyramidal neurons.

### Excitatory and Inhibitory Synaptic Inputs to Dendrites and Soma of Different Types of Neurons

**Figure 3:**
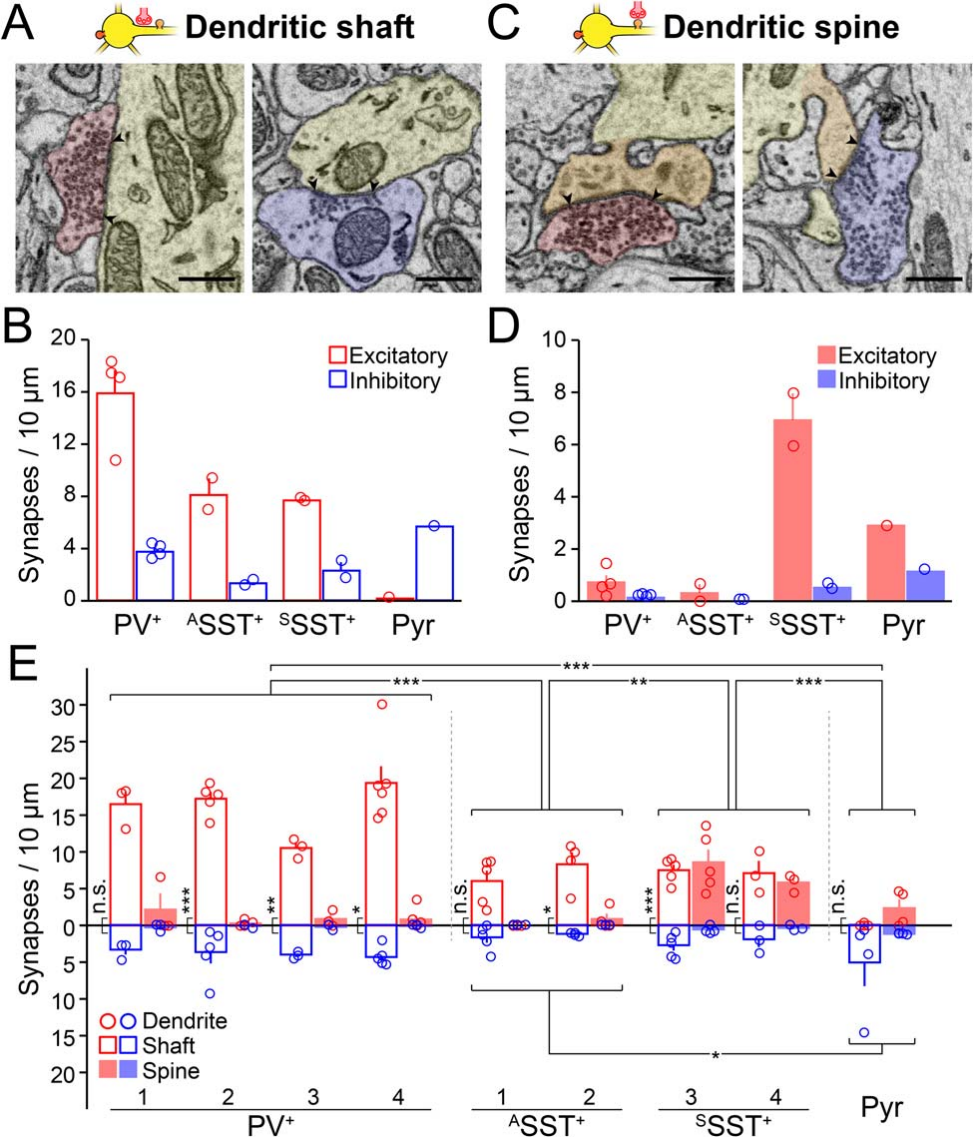
Excitatory and inhibitory synapses on dendrites. (*A*) Representative SBEM images showing excitatory (red) and inhibitory (blue) pre-synaptic inputs to the dendritic shaft (yellow). Scale bars, 500 nm. (*B*) The density of excitatory (red) and inhibitory synapses (blue) on dendritic shaft of different neuronal types. Bars, means ± SEM; open circles, individual cells. (*C* and *D*) Same as (*A* and *B*) but for synapses on dendritic spines (orange). (*E*) The density of excitatory and inhibitory synapses on individual neurons. Circles, the number of excitatory (red) and inhibitory (blue) synapses per 10 μm of individual dendritic shafts (open bars) and spines (closed bars); bars, means ± SEM. Number of excitatory and inhibitory synapses across cell types, ^*^P < 0.05, ^**^P < 0.01, ^***^P < 0.001, ANOVA test with Bonferroni correction; number of excitatory and inhibitory synapses on dendrites of each neuron, ^*^P < 0.05, ^**^P < 0.01, ^***^P < 0.001, n.s., not significant, paired *t*-test or Wilcoxon signed-rank test depending on the data distribution.

**Figure 4:**
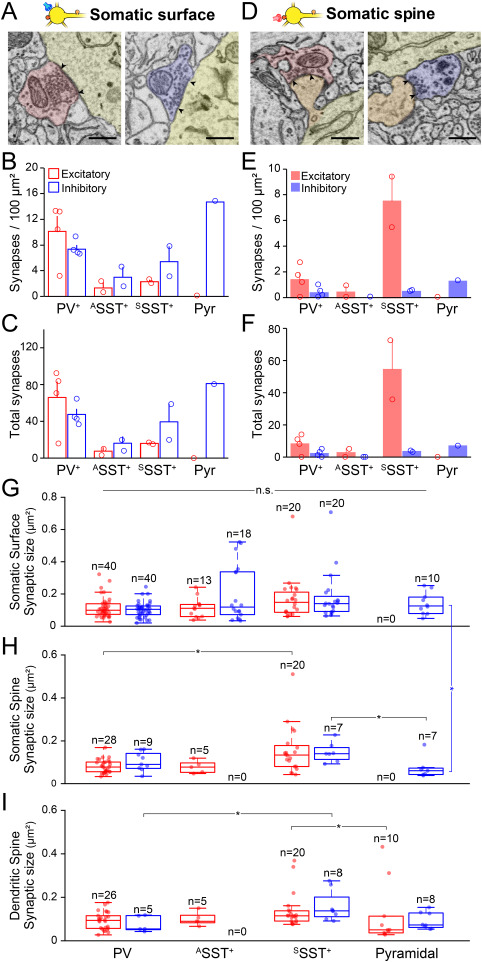
Excitatory and inhibitory synapses on soma. (*A*) Two representative SBEM images showing excitatory (red) and inhibitory (blue) pre-synaptic inputs to the somatic surface. Scale bars, 500 nm. (*B*) The density of excitatory (red) and inhibitory synapses (blue) on the somatic surface of different neuronal types. Bars, means ± SEM; open circles, individual cells. (*C*) Total number of excitatory (red) and inhibitory (blue) on the somatic surface of different neuronal types. Bars, means ± SEM; open circles, individual cells. (*D*–*F*) Same as (*A*–*C*) but for synapses on somatic spines (orange in *D*). (*G*–*I*) Box plots, the size of excitatory (red) and inhibitory (blue) synapses (μm^2^) on the somatic surface (*G*), somatic spine (*H*), and dendritic spine (*I*) of different neuronal types. Circles, individual excitatory and inhibitory synapses. Comparison across cell types (horizontal lines) or within cell type (vertical lines), ^*^P < 0.05, n.s., not significant; Kruskal–Wallis test with Bonferroni correction.

We next counted the number of synapses formed on the soma and dendrites in different types of neurons (a total 1974 synapses in 9 neurons) and classified the synapses as excitatory (E) or inhibitory (I) based on their pre- and post-synaptic ultrastructures (Eccles 1964; Korogod et al. 2015; Huang et al. 2018). To understand how the E/I synaptic inputs are formed in distinct cell types depending on their locations, we examined the number of excitatory and inhibitory synapses on the dendrites (shaft vs. spines; Fig. 3) and the soma (surface vs. spines; Fig. 4).

First, the synapses that formed on the perisomatic dendrites of inhibitory neurons were mostly excitatory (Fig. 3*A*–*E*: excitatory synapses per 10 μm of dendrite, on the spine, 0.73 ± 0.26, 0.33 ± 0.33, and 6.95 ± 1.01, on the shaft, 15.90 ± 1.75, 8.11 ± 1.22, and 7.69 ± 0.11; inhibitory synapses per 10 μm of dendrite, on the spine, 0.16 ± 0.02, 0.00 ± 0.00, and 0.52 ± 0.11, on the shaft, 3.75 ± 0.27, 1.29 ± 0.22, and 2.30 ± 0.66; for PV^+^, ^A^SST^+^, and ^S^SST^+^ neurons, respectively). On the other hand, the pyramidal neuron received prominent inhibitory inputs on the dendritic shaft while receiving slightly more excitatory inputs on the spines (Fig. 3*B*,*D*; excitatory synapses per 10 μm of dendrite; on the shaft, 0.14, on the spine, 2.90; inhibitory synapses per 10 μm of dendrite, on the shaft, 5.65, on the spine, 1.16). Excitatory inputs to the perisomatic dendrites were significantly more numerous on PV^+^ and ^S^SST^+^ neurons compared to on ^A^SST^+^ neurons and the pyramidal neuron. The pyramidal neuron received more inhibitory inputs on dendrites compared with the ^A^SST^+^ neurons (Fig. 3*E*).

Next, we measured synapses on the soma and found distinct features across cell types (Fig. 4*B*–*F*). PV^+^ neurons received more excitatory than inhibitory synaptic inputs on both the surface and the spines of the soma (Fig. 4*B*,*E*: PV^+^, excitatory synapses per 100 μm^2^ soma, on the surface, 9.96 ± 2.38, on the spine, 1.40 ± 0.57; inhibitory synapses per 100 μm^2^ soma, on the surface, 7.43 ± 0.62, on the spine, 0.40 ± 0.21). On the other hand, all SST^+^ neurons received more inhibitory synaptic inputs than excitatory synaptic inputs on the somatic surface, while ^S^SST^+^ neurons received much stronger excitatory inputs on the somatic spines (Fig. 4*B*,*E*: ^A^SST^+^, excitatory synapses per 100 μm^2^ soma, on the surface, 1.40 ± 0.86, on the spine, 0.45 ± 0.45; inhibitory synapses per 100 μm^2^ soma, on the surface, 2.99 ± 1.54, on the spine, 0.00 ± 0.00; ^S^SST^+^, excitatory synapses per 100 μm^2^ soma, on the surface, 2.27 ± 0.30, on the spine, 7.51 ± 2.06; inhibitory synapses per 100-μm^2^ soma, on the surface, 5.38 ± 2.35, on the spine, 0.49 ± 0.03). Pyramidal neurons received only inhibitory synaptic inputs on the soma either on the surface or on the spines (Fig. 4*B*,*E*: Pyr, excitatory synapses per 100 μm^2^ soma, on the surface, 0.00, on the spine, 0.00; inhibitory synapses per 100 μm^2^ soma, on the surface, 14.81, on the spine, 1.28). To further quantify differences in synaptic input across cell types, we also compared the total number of synapses on each soma (Fig. 4*C*,*F*). In the case of the ^A^SST^+^ neurons, serial EM images did not cover the soma completely. Thus, we estimated the total number of synapses assuming that the soma is an ellipsoid (Fig. S1). We also found the same result in the total amounts of excitatory and inhibitory synaptic inputs to the soma as we saw in the synaptic density (Fig. 4*C*,*F*: PV^+^, total excitatory synapses per soma, on the surface, 66.00 ± 17.27, on the spine, 8.25 ± 3.01; total inhibitory synapses per soma, on the surface, 47.50 ± 6.08, on the spine, 2.25 ± 1.11; ^A^SST^+^, total excitatory synapses per soma [estimated], on the surface, 7.53 ± 4.22, on the spine, 2.76 ± 2.76; total inhibitory synapses per soma [estimated], on the surface, 16.16 ± 7.34, on the spine, 0.00 ± 0; ^S^SST^+^, total excitatory synapses per soma, on the surface, 16.00 ± 1.00, on the spine, 54.50 ± 18.50; total inhibitory synapses per soma, on the surface, 39.50 ± 19.50, on the spine, 3.50 ± 0.50; Pyr, total excitatory synapses per soma, on the surface, 0, on the spine, 0; total inhibitory synapses per soma, on the surface, 81, on the spine, 7). Our data on the pyramidal neuron support previous EM studies showing that the somatic spines of the pyramidal neurons receive strong inhibitory synaptic inputs (Parnavelas et al. 1977; Defelipe and Farinas 1992). Thus, the E/I input ratio estimated from the number of synapses on the perisoma differs between cell types, and inhibitory neurons received stronger excitatory inputs to the perisoma than did the pyramidal neuron.

Previous reports have shown that the size of synaptic junctions can represent the amount of synaptic currents (Kubota et al. 2015). To further estimate the E/I perisomatic inputs on different types of neurons, we measured the size of synapses that were located at different compartments of the perisoma across the neuronal types (Fig. 4*G*–*I*). Overall, there were no clear differences between sizes of excitatory and inhibitory synapses on each neuronal type (Fig. 4*G*–*I*; comparison between red and blue bars as a pair). Interestingly, when we compared synaptic size across cell types, the size of both excitatory and inhibitory synapses on the spines of ^S^SST^+^ neurons was slightly bigger than that of PV^+^ neurons or the pyramidal neuron (Fig. 4*H*,*I*). Although all inhibitory neurons showed similar synaptic sizes regardless of synaptic location, the size of synapses on the somatic surface of the pyramidal neuron was larger than that on the spines (Fig. 4*G*,*H*). Together, our data indicate that the E/I ratio estimated from the number of synaptic inputs on each neuronal type is likely a useful measure and that excitatory synaptic inputs to the ^S^SST^+^ neurons and inhibitory inputs to the pyramidal neuron can be even stronger than to other cell types.

### Excitatory and Inhibitory Synaptic Inputs to Spines

The spine is an important receiver of synaptic inputs on the post-synaptic membrane. The spine’s structure helps determine the effect of the pre-synaptic release on changing post-synaptic membrane potential. We therefore examined the length and the volume of spines across cell types and the number of synapses formed on each spine of the four different types of neurons (37 spines from 4 PV^+^ neurons, 5 spines from two ^A^SST^+^ neurons, 104 spines from two ^S^SST^+^ neurons, and 41 spines from one pyramidal neuron). We did not categorize the spines as previously done (Harris et al. 1992; Risher et al. 2014; Foggetti et al. 2019) because the spine length, volume, head-to-neck, and length-to-head ratios appeared continuous (Arellano et al. 2007).

**Figure 5:**
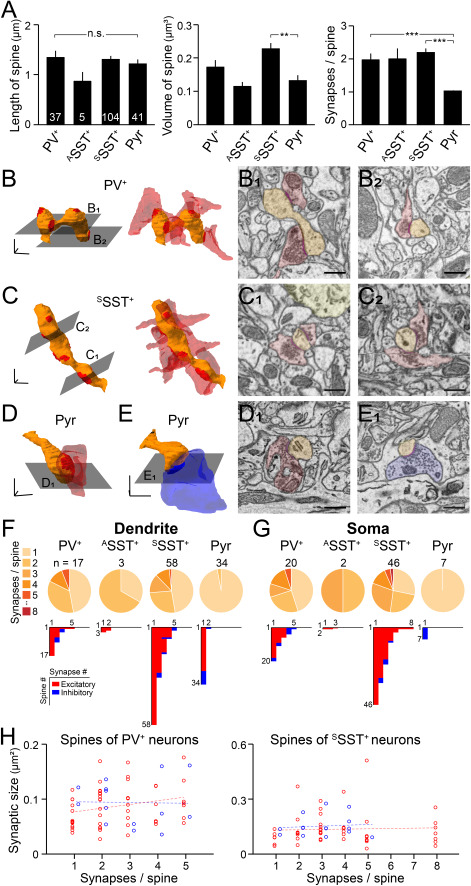
Diversity in synapse organization on the spine of different types of neurons. (*A*) Spine length (left), volume (middle), and the number of synapses (right) on each spine of classified cell types: PV^+^ neurons (*N* = 4, *n* = 37); ^A^SST^+^ neurons (*N* = 2, *n* = 5); ^S^SST^+^ neurons (*N* = 2, *n* = 104); and pyramidal neurons (Pyr, *N* = 1, *n* = 41). Bars, means ± SEM. Note that ^S^SST^+^ neurons have larger volumes and more synapses on the spine. ^**^P < 0.01, ^***^P < 0.001; n.s., not significant; Kruskal–Wallis test with Bonferroni correction. (*B*–*E*) 3D structure of a representative spine in each cell type (orange) with excitatory (red) and inhibitory (blue) pre-synaptic contacts. 3D-scale bars, 500 nm. (*B*) The spine of a PV^+^ neuron forming five excitatory synapses. (*C*) The spine of an ^s^SST^+^ neuron forming eight excitatory synapses. (D) The spine of a pyramidal neuron forming an excitatory synapse. (E) The spine of a pyramidal neuron forming an inhibitory synapse. (*B*_1_–*E*_1_) Representative SEM images of spines (orange) and excitatory (red) or inhibitory (blue) pre-synaptic boutons on the spine. Scale bars, 500 nm. (*F* and *G*) Top pie charts, number of synapses per spine on dendrites (*F*) and soma (*G*) of each neuronal type. Bottom histograms, number of excitatory (red) and inhibitory (blue) synapses on individual spines. (H) Scatter plots of synaptic sizes (μm^2^) and the number of synapses per a spine of PV^+^ (*N* = 4, *n* = 68) and ^S^SST^+^ (*N* = 2, *n* = 65) neurons. Red, excitatory synapses; blue, inhibitory synapses; dots, individual synapses; lines, linear regression of the scatter plots. Note that synaptic sizes of individual synapses were similar across spines with different numbers of synaptic inputs.

Although the mean length of spines did not differ significantly between cell types (Fig. 5*A*, left: 1.34 ± 0.13, 0.87 ± 0.18, 1.31 ± 0.07, and 1.22 ± 0.09 μm for PV^+^, ^A^SST^+^, ^S^SST^+^, and the pyramidal neurons, respectively), the volume of the spines of ^S^SST^+^ neurons was significantly larger than that of the spines of the pyramidal neuron (Fig. 5*A*, middle: 0.17 ± 0.02, 0.11 ± 0.01, 0.23 ± 0.02, and 0.13 ± 0.02 μm^3^ for PV^+^, ^A^SST^+^, and ^S^SST^+^ neurons and the pyramidal neuron, respectively). Furthermore, unlike the spines of the pyramidal neuron, which received mostly one synapse per spine (Leranth et al. 2003), spines on the GABAergic neurons received multiple synaptic inputs on a single spine (Fig. 5*A*, right: 1.97 ± 0.20, 2.00 ± 0.32, 2.20 ± 0.13 and 1.02 ± 0.02 synapse number per spine for PV^+^, ^A^SST^+^, and ^S^SST^+^ and the pyramidal neurons, respectively). In particular, the spines of ^S^SST^+^ neurons carried significantly more synapses, each with a larger volume than the spines of the pyramidal neuron (*P* < 0.01 for the volume of spines and *P* < 0.001 for the number of synapses per spine), despite their similar length.

We then examined the distribution of synapses per spine in GABAergic neurons; PV^+^ and ^S^SST^+^ neurons showed up to eight synapses per spine (Fig. 5*B*, C, *F*, and *G*). The distribution of the synapses, which were mostly excitatory, contrasted with that found on the pyramidal neuron, which showed only one synapse per spine (Fig. 5*D*–*G*; Movie S3). On the other hand, ^A^SST^+^ neurons received at most only a few excitatory synapses per spine (Fig. 5*F*,*G*). The synapses on the spines in the pyramidal neuron tended to be inhibitory on the soma and excitatory on the dendrites (Fig. 5*F*,*G*). Although spines of PV^+^ and ^S^SST^+^ neurons have up to 8 synapses, we found no correlation between the number of synapses and the size of synapses, and the size of synapses on the spines that have multiple synapses did not differ from the size of synapses on the spines that have only one synapse (Fig. 5*H*). Collectively, our data indicate that the spines of GABAergic neurons differ structurally from those of a pyramidal neuron and integrate multiple synaptic inputs into one spine.

### Structural Organization of Excitatory and Inhibitory Pre-Synaptic Boutons on Different Types of Neurons

**Figure 6:**
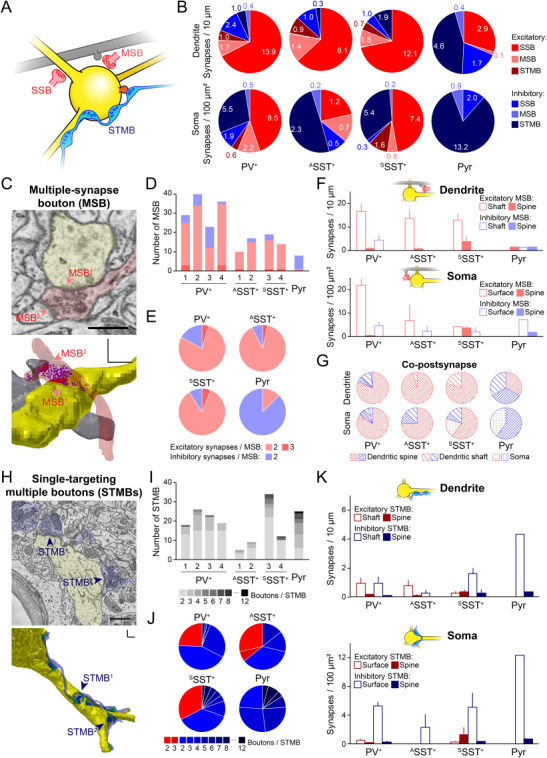
Multiple-synapse boutons and single-targeting multiple boutons on different types of neurons. (*A*) Schematic of a single-synapse bouton (SSB, red), a multiple-synapse bouton (MSB, red) and single-targeting multiple boutons (STMBs, blue) on an identified neuron (yellow). Gray, an unidentified co-postsynaptic neuron of the MSB. (*B*) The number of synaptic inputs on the dendrites and soma of each cell type. Primary color, single-synapse boutons; light color, MSBs; dark color, STMBs; red, excitatory; blue, inhibitory. (*C*) Top, a representative SBEM image showing an MSB (red), forming two synapses (red arrows) on the identified neuron (yellow, SST^+^), and an unidentified neuron (gray). Scale bar: 500 nm. Bottom, 3D reconstruction of the MSB in the top. Yellow, dendrite; red, excitatory synaptic contact; red shade, excitatory pre-synaptic bouton; gray, another post-synaptic target (a dendritic spine of an unidentified neuron); magenta dots, excitatory synaptic vesicles. 3D-scale bars, 500 nm. (*D*–*E*) The number of synapses (double or triple) formed by each MSB, including the synapse on the reconstructed neuron in individual neurons (bars) and in different types of neurons (pie charts). Red, excitatory boutons; blue, inhibitory boutons. Note that the MSBs are mostly excitatory inputs in all inhibitory neurons but not in the pyramidal neuron. (*F*) The number of MSBs on dendrites (top) or on soma (bottom) of the reconstructed neurons. (*G*) The proportion of other post-synaptic targets of the MSBs found in either dendrites (top) or soma (bottom) of the reconstructed neurons. (*H*) Same as (*C*) but for an STMB (blue) making at least six inhibitory synaptic contacts (blue and blue arrows). Green dots, inhibitory synaptic vesicles. Scale bar, 1 μm; 3D-scale bars, 1 μm. (*I*) The number of STMBs with different numbers of boutons that were found in each reconstructed neuron. (*J*) The proportion of excitatory axons (red) and inhibitory axons (blue), which formed STMBs on different types of neurons. The color and number represent the number of boutons per STMB. (*K*) Bars, same as (*F*) but for STMBs. Note that most STMBs are inhibitory synapses on the somatic surface. Bars, means ± SEM.

We examined structural properties of individual pre-synaptic boutons and traced other synaptic contacts made by these axons. Many of the axons made a synapse on the target neuron by forming a single-synapse bouton (SSB; Fig. 6*A*). Interestingly, some boutons formed multiple (mostly dual) synapses on different neurons, one on the identified perisoma and others on nearby neurons. We categorized these synaptic boutons as MSBs (Fig. 6*A*). Furthermore, some synaptic boutons were from one axon that made multiple synapses in a series on the same target neuron. We classified those as STMBs (Fig. 6*A*).

Most of the MSBs were excitatory synapses, and up to 36.6% of total excitatory synapses on the perisoma of inhibitory neurons were the MSBs (Fig. 6*B*–*F*; light red). On the other hand, we rarely observed excitatory MSBs making synapses on the pyramidal neuron. Instead, a small subset of inhibitory synapses on the pyramidal neuron was MSBs (Fig. 6*B*–*F*; light blue). Although MSBs have been identified previously in the mammalian cortex (Tweedle and Hatton 1984; Sorra and Harris 1993; Knott et al. 2006), their post-synaptic identities are not yet fully understood. We found that most MSBs formed synapses on the perisomatic shaft rather than the spines of identified GABAergic neurons (Fig. 6*F*). Among the co-postsynaptic structures we examined, the dendritic spines of unidentified neighboring neurons were the most frequent targets of the excitatory MSBs (Fig. 6*G*). In contrast, inhibitory MSBs often formed synapses on the dendritic shaft of other neurons, highlighting the differences in the post-synaptic structures between excitatory and inhibitory MSBs (Fig. 6*G*).

On the other hand, STMBs accounted mainly for inhibitory synapses, and individual boutons in the STMBs were rarely MSBs (% of MSBs in STMBs, 7.01 ± 1.81, 8.39 ± 0.70, 2.04 ± 2.04, and 2.91 for PV^+^, ^A^SST^+^, and ^S^SST^+^ neurons and the pyramidal neuron, respectively). Up to eight boutons from an STMB were found on a GABAergic interneuron and up to 12 were found in the pyramidal neuron (Fig. 6*H*–*J*). Across all cell types, more than 70% of somatic inhibitory synapses were from STMBs (Fig. 6*B*), suggesting that these STMBs are from basket cells that exert strong perisomatic inhibition (Karube et al. 2004). In contrast, excitatory STMBs were rarely found across all neuronal types, although a subset of STMBs in inhibitory neurons were excitatory synapses (Fig. 6*K*). Collectively, these data suggest that V1 microcircuits are constructed to promote inhibition in the network, particularly strong perisomatic inhibition by STMBs on pyramidal neurons.

## Discussion

Previous studies have tried to classify neuronal types based on morphological characteristics such as spines (Karube et al. 2004; Kawaguchi et al. 2006; Scheuss and Bonhoeffer 2014; Sancho and Bloodgood 2018). However, these studies did not examine the difference in the structure of synaptic inputs. In this study, we visualized the 3D structure of synaptic inputs to proximal parts of genetically identified neurons using SBEM and, further, demonstrated that in distinct neuronal types, including PV^+^, SST^+^, and pyramidal neurons, pre-synaptic structures have unique features. We focused on the perisomatic inputs, as these have the most powerful impact on modulating neuronal membrane potential and generating action potential outputs. We revealed that distinct neuronal types have different combinations of excitatory and inhibitory synaptic inputs on the perisomatic membrane. The properties of such input structures may help explain the cell-type-specific function of V1 neurons *in vivo* (Atallah et al. 2012; Lee et al. 2012; Wilson et al. 2012).

We identified the excitatory and inhibitory synaptic inputs based on the structural features of the synapses found in the EM images: the symmetry between pre- and post-synaptic membranes and the shape of synaptic vesicles (type I vs. type II synapses). Although some of the synapses might be neuromodulatory, such as cholinergic or noradrenergic synapses (Kim et al. 2016), there would be fewer noncanonical synapses than canonical glutamatergic and GABAergic synapses in the cortex. Nevertheless, to confirm chemical properties of synaptic inputs that we morphologically defined in the V1, future studies tracing chemically or genetically identified axons and their synaptic targets are necessary.

The thickest dendrites emerging from the soma were those carrying spines. ^A^SST^+^ neurons have thinner dendrites than ^S^SST^+^ neurons. These data support earlier reports showing that spine formation thickens and stabilizes dendrites (Koleske 2013). We often failed to identify axons, as our SBEM images covered only a limited distance (11.91 ± 0.85 μm) from the soma. Within this range, the structures on the axons and the dendrites may not differ much; spines and synaptic inputs were found near the soma of both axons and dendrites (Fig. 2*A*). In this study, however, we identified two unique protrusions from the soma of cortical neurons: cilia and spines. First, all the neurons that we examined had one typical ciliary protrusion from the soma. The somatic cilia showed uniform thickness and length, as well as microtubular and centriolar structures (Guemez-Gamboa et al. 2014). We did not find any particular orientation of the ciliary protrusions from the soma or any synaptic inputs on them. Cilia are known to be important for cellular division and migration (Guemez-Gamboa et al. 2014), and many G-protein-coupled receptors are enriched in them (Guadiana et al. 2013). As we found that every neuron has cilia, it will be interesting to study the function of cilia in adult V1 neurons *in vivo*.

Next, we found that the spiny neurons in the V1 had a significant number of spines on the soma. This observation was striking and more prominent in ^s^SST^+^ neurons (Fig. 2*G*). Spines of the soma have been found in other brain areas and several neuronal types (Bundman et al. 1994; Wenzel et al. 1994; Shoop et al. 2002), but their function in cortical neurons has not yet been fully identified. Future studies are necessary to understand the difference or similarity between excitatory synaptic inputs to somatic spines and to dendritic spines. Inhibitory synapses on the dendritic spines of pyramidal neurons are known to induce focal inhibition (Chiu et al. 2013), and future studies are required to understand whether the inhibition on the perisomatic spines can be local as the volume of the soma is much larger than that of the distal dendrites.

It is widely known that the functional diversity of SST^+^ neurons stems from their heterogeneity in the cortex (Yavorska and Wehr 2016). A recent high-throughput study on interneurons in the primary visual cortex showed that SST^+^ neurons in the layer 2/3 can be divided into three morphologically distinct types: Martinotti cells, which have a multipolar appearance and a dominant axonal arbor in the layer 1; neurons with a bitufted appearance; neurons with basket-cell anatomy and fast-spiking phenotype (Jiang et al. 2015). Furthermore, SST^+^ neurons in the mouse V1 can be divided into two functional classes: those that are tuned selectively or broadly to orientations (Kerlin et al. 2010). We also found two distinct classes of SST^+^ neurons in the layer 2/3 of V1, according to the presence of spines: ^S^SST^+^ and ^A^SST^+^. It is highly plausible that our ^S^SST^+^ neurons are the Martinotti neurons, which are known to be fluorescently labeled in the GIN mouse (Ma et al. 2006; Fanselow et al. 2008). Martinotti cells are known to be spinier than other interneurons (Kawaguchi et al. 2006) and show sharply tuned orientation selectivity (Ma et al. 2010). In contrast, ^A^SST^+^ neurons that we found might be the bitufted or the basket cell with broad-tuning properties. Future studies are required to understand the cell types of ^A^SST^+^ neurons.

Spines on the inhibitory neurons differed structurally from typical mushroom-like spines. They showed complicated 3D structures that can integrate multiple synaptic inputs. Spines of the pyramidal neuron show orientation selectivity like the neuron’s selective outputs (Jia et al. 2010). It will be interesting to examine whether the spines of interneurons show a similarly selective response to the visual stimuli, despite receiving multiple inputs to a single spine. Previous studies have classified the structure of spines based on the size and length of heads and necks and found that those features correlate with the longevity and plasticity of the spine (Holtmaat et al. 2005; Holtmaat et al. 2006; Knott et al. 2006). For instance, the spines of PV^+^ neurons in the mouse hippocampus were often stubby or thin, and thus unstable, compared with the more common mushroom-type spines (Foggetti et al. 2019). The divergent spine structures that we found in interneurons suggest that the spines of inhibitory neurons might be more plastic than typical spines in the pyramidal neurons. Indeed, it has been shown that the spines of interneurons in V1 show rapid structural changes in response to sensory deprivation (Keck et al. 2011). The plasticity of V1 neurons may help explain why the number of spines that we counted in the Martinotti neuron (GIN neuron) was double that found in the frontal cortex of the rat (Kawaguchi et al. 2006). It is also possible that the excitatory inputs to the spines of ^S^SST^+^ neurons may play a key role in synaptic plasticity during learning (Makino and Komiyama 2015).

**Figure 7:**
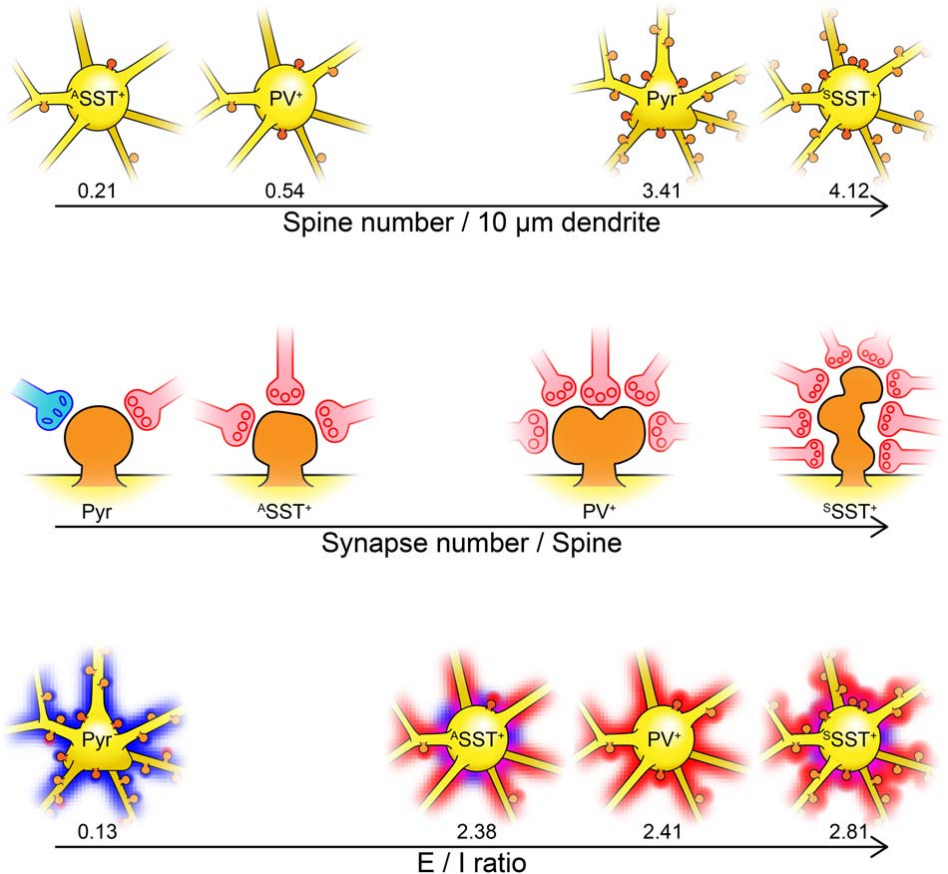
Organization of synaptic inputs for various cell types in layer 2/3 mouse V1. In this study, we found that the synapse organization in the proximal parts of PV^+^ and SST^+^ GABAergic interneurons and the pyramidal neuron were distinguished by their spine density (top), synapse number per spine (middle), and the ratio of excitatory and inhibitory synapses (bottom). Yellow, soma and dendrites; orange, spines; red shade, excitatory inputs; blue shade, inhibitory inputs.

Finally, our study suggests that excitation and inhibition balance each other in the cell-type-specific ultrastructures of V1 neurons, according to the specific regimes each uses to organize synaptic inputs near the soma (Fig. 7). First, excitatory neurons receive more inhibitory synaptic inputs and inhibitory neurons receive more excitatory inputs on the perisomatic membrane. In particular, PV^+^ neurons, which are fast-spiking and exert strong perisomatic inhibition on nearby excitatory neurons (Freund and Katona 2007; Ascoli et al. 2008), received massive excitatory synapses on their perisoma. This synaptic input structure indicates that excitatory neurons in V1 activate inhibitory neurons. Second, inhibitory synaptic inputs, but not excitatory synaptic inputs, to the perisoma were predominantly STMBs and manifested strong perisomatic inhibition (Tremblay et al. 2016). Finally, excitatory synaptic inputs to the inhibitory neurons were often MSBs; these formed other excitatory synapses on the dendritic spines of unidentified neurons (possibly on the spiny excitatory neurons that make up the majority of neurons in the cortex). All of these structural features are consistent with a cortical network that balances excitation with inhibition.

We examined local structure of V1 circuits from fixed brain tissue, and future studies are required to understand the function of these structures and the origin of synaptic inputs. For example, the MSB structure of excitatory synapses on inhibitory neurons may drive temporally synchronized inputs to both distal dendrites of excitatory neurons and proximal dendrites of inhibitory neurons to generate strong feed-forward inhibition. Alternatively, the MSBs may represent less-mature synaptic structures (Toni et al. 2007) or the synapses that formed after learning or experiences (Lee et al. 2013; Yang et al. 2016; Kim et al. 2019). MSBs on the V1 neurons may originate from the thalamus, since it has been shown that thalamic inputs to the V1 often form MSBs on V1 neurons (Friedlander et al. 1991; Jones et al. 1997). Overall, our findings suggest that excitatory and inhibitory inputs in V1 use different strategies to develop synapses on the perisomatic region of distinct types of neurons. Future studies are required to understand functional aspects of the particular structure of synaptic inputs *in vivo*.

## Supplementary Material

Supplementary_Data_bhaa378

Supplementary_Video1_bhaa378

Supplementary_Video2_bhaa378

Supplementary_Video3_bhaa378
