# Supplementary material for: 3D Ultrastructure of Synaptic Inputs to Distinct GABAergic Neurons in the Mouse Primary Visual Cortex
Source: Cereb Cortex. 2020 Dec 22;31(5):2610–24. doi: 10.1093/cercor/bhaa378 (PMC8023854; doi:10.1093/cercor/bhaa378)
Supplement: Supplementary_Data_bhaa378 [file supplementary_data_bhaa378.docx]

**Supplementary Figure 1. Estimated 3D structure of the soma of ^A^SST^+^ neurons. (A)** 3D reconstruction of ^A^SST^+^ neurons by assuming that the soma is an ellipsoid. Note that the gray part of the soma was drawn based on the estimation of an ellipsoid across the somatic surface. **(B)** The volume of the soma from 9 neurons. Yellow, quantification based on EM images; gray, quantification based on estimation. **(C)** The surface area of the soma from 9 neurons. Yellow, quantification based on EM images; gray, quantification based on estimation.

**Supplementary Movie 1. Correlative SBEM for 3D reconstruction of a fluorescence-labeled (SST^+^) neuron.**

**Supplementary Movie 2. Three types of protrusions from the identified soma.**

**Supplementary Movie 3. Input structures on the spines of different cell-types.**
